# Temporal neural coupling: reconciling the Bobath concept with task-oriented training for post-stroke upper limb recovery—a perspective

**DOI:** 10.3389/fneur.2026.1857483

**Published:** 2026-07-09

**Authors:** Dong Guo, He Zhang, Dan Zou, Zhao-min Ding

**Affiliations:** 1 Department of Rehabilitation, Hongqi Hospital Affiliated to Mudanjiang Medical University, Mudanjiang, China; 2 Department of Central Sterile Supply, Hongqi Hospital Affiliated to Mudanjiang Medical University, Mudanjiang, China; 3 Department of Nursing Care, Hongqi Hospital Affiliated to Mudanjiang Medical University, Mudanjiang, China; 4 Second Ward of Cardiology Department, Hongqi Hospital Affiliated to Mudanjiang Medical University, Mudanjiang, China

**Keywords:** Bobath concept, closed-loop neuromodulation, neuroplasticity, precision neurorehabilitation, stroke rehabilitation, task-oriented training, temporal neural coupling, upper extremity

## Abstract

Post-stroke neurorehabilitation has long been shaped by a binary opposition between the Bobath concept and task-oriented training. The former emphasizes inhibition of aberrant patterns and facilitation of normative movement, while the latter prioritizes high-intensity, goal-directed repetition that may inadvertently reinforce compensatory strategies. This dichotomy persists largely because mechanistic frameworks capable of explaining their potential complementarity remain underdeveloped. This perspective article advances the hypothesis of temporal neural coupling. We propose that manual facilitation and postural preparation within the contemporary Bobath concept serve to optimize the spike-timing precision of descending corticospinal commands and the recruitment fidelity of spinal motor networks. This preparatory modulation establishes a low-entropy motor baseline and opens a permissive temporal window for inducing use-dependent long-term potentiation during subsequent task-specific practice. We argue that these modalities should not be viewed as discrete, sequentially administered phases. Rather, they constitute dynamically time-locked, synergistic components of a unified intervention strategy. The perspective article further outlines a future paradigm grounded in closed-loop neurofeedback, wherein real-time neurophysiological monitoring guides the precise, state-dependent orchestration of plasticity tailored to each patient’s recovery trajectory.

## 1 Introduction

Restoring upper limb function after stroke remains a formidable clinical challenge. Epidemiological data indicate that approximately 50–80% of acute stroke survivors exhibit upper extremity motor deficits, and nearly 50% continue to experience functional impairment of the affected limb at 6 months post-stroke (1, 2). The pathophysiology underlying this persistent disability involves a cascade of maladaptive changes, including loss of descending cortical inhibition, aberrant spinal circuit reorganization, and the behavioral phenomenon of learned non-use (3). Despite advances in early intervention, recovery often reaches a plateau, with existing behavioral protocols demonstrating limited efficacy in restoring distal dexterity following severe corticospinal tract injury (4).

Current practice is marked by a conceptual tension between two dominant paradigms. Task-oriented training employs repetitive, goal-directed practice to drive use-dependent plasticity (5). However, without precise kinematic monitoring, patients frequently accomplish tasks through maladaptive compensatory movements—such as excessive shoulder elevation and trunk displacement (6). This reliance on alternative motor pathways risks reinforcing aberrant synergies and impeding the emergence of isolated joint control (6). Conversely, the Bobath concept aims to inhibit abnormal tone and facilitate more normative movement patterns through therapist-guided sensory input (7). While clinically intuitive, this approach has drawn criticism for potential over-reliance on passive handling, which may attenuate the generation of sensory prediction errors essential for active motor learning and synaptic consolidation (8). A notable paradox thus arises: although combining Bobath preparation with task-oriented training appears theoretically synergistic, multiple meta-analyses report no significant long-term differences in functional outcomes (9). This null finding likely reflects a failure to stratify participants by key neurobiological variables—such as corticospinal tract integrity or intracortical inhibition—rather than a true absence of differential effect (10) (Table 1).

**Table 1:** Conceptual and neurophysiological comparison of rehabilitation paradigms.

Domain	Bobath concept	Task-oriented training	Temporal coupling framework (proposed)
Core principle	Tone inhibition and facilitation	Repetition and goal-directed learning	Timing-dependent synergy
Neurophysiology	Sensory modulation, afferent reweighting	Reward-based plasticity	STDP + signal-to-noise optimization
Strength	Improves movement quality	Enhances motor learning	Maximizes plasticity efficiency
Limitation	Passive, therapist-dependent	Risk of compensation	Requires monitoring system
Temporal role	Preparation phase	Execution phase	Dynamically integrated
Plasticity mechanism	Noise reduction	Reinforcement learning	Coupled Hebbian plasticity
Clinical implication	Normalize baseline	Drive performance	Personalized adaptive rehab

STDP, Spike-Timing-Dependent Plasticity.

The central bottleneck addressed in this perspective is not simply insufficient training intensity or inadequate movement quality, but rather the failure to align rehabilitative input with transient neurophysiological states that are permissive for plastic change. Contemporary rehabilitation paradigms typically emphasize either movement normalization (Bobath concept) or intensive task repetition (task-oriented training); however, neither framework explicitly addresses when the nervous system is most receptive to adaptive synaptic modification (11, 12). We therefore hypothesize that rehabilitation efficacy may depend on the temporal synchronization of preparatory state optimization and reinforcement-based motor learning. The temporal neural coupling framework is proposed as a conceptual mechanism through which these otherwise separate therapeutic processes may interact. Addressing this impasse demands a mechanistic reinterpretation of how these interventions interact at the level of the motor apparatus.

Importantly, the mechanistic framework proposed in this article should be interpreted as a hypothesis-generating model, not as an empirically validated neurophysiological pathway. Several key constructs—including “motor noise reduction,” “low-entropy states,” and “temporal coupling”—are employed here as operational metaphors (13, 14). Although grounded in established motor control theory, these metaphors require further empirical validation (14, 15). In this context, “low entropy” specifically denotes reduced trial-to-trial variability in muscle activation patterns, which can be approximated using metrics such as the coefficient of variation in surface electromyographic signals or the temporal dispersion of motor unit recruitment (16–18).

**Figure 1:**
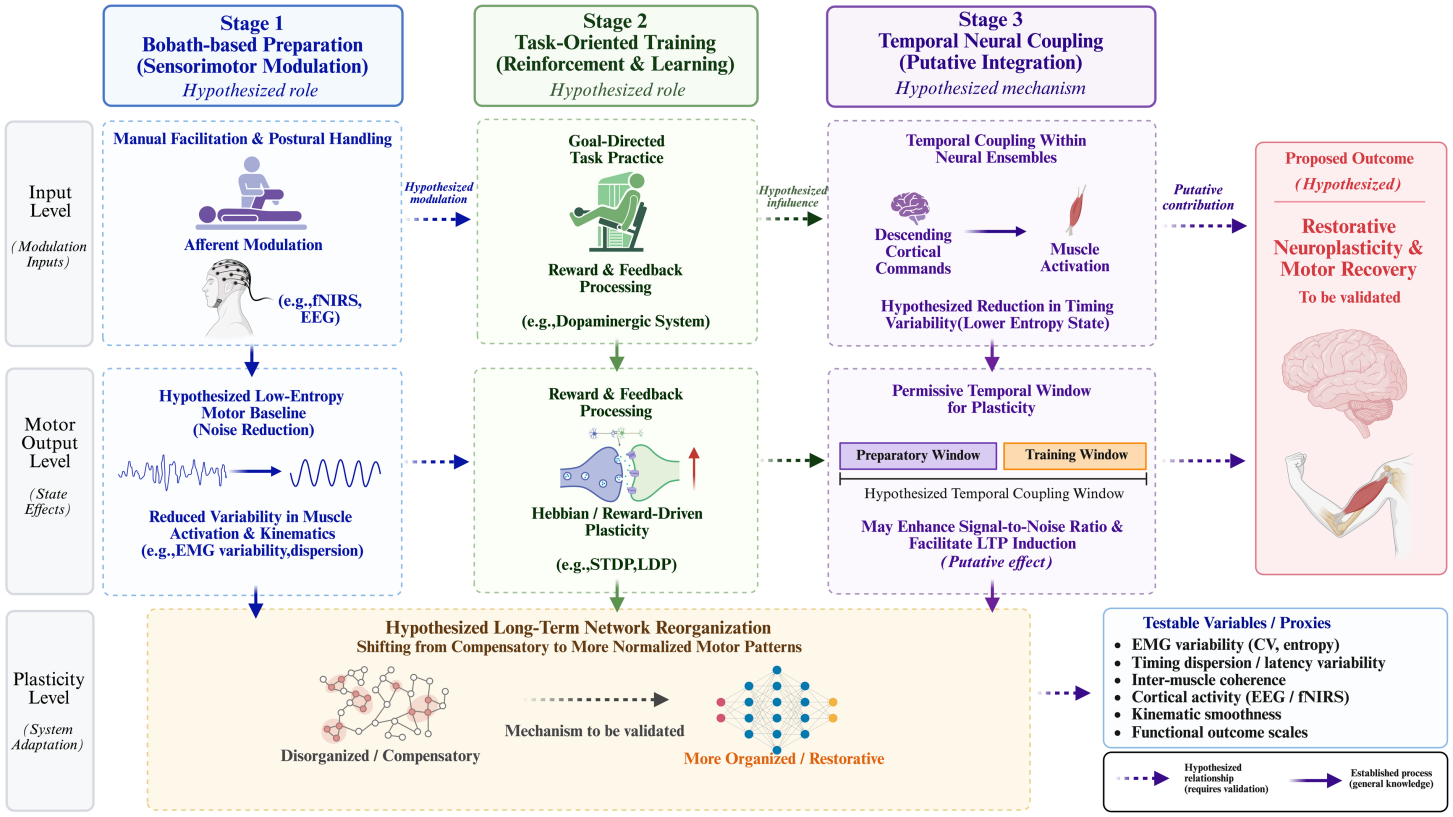
Conceptual and hypothesis-generating framework of temporal neural coupling. The diagram illustrates proposed interactions among sensorimotor modulation, temporal organization of motor output, and reinforcement-driven plasticity. Dashed arrows represent hypothesized relationships that require empirical validation. This figure is intended to provide a conceptual model rather than a definitive or experimentally confirmed mechanism.

Building on this conceptual foundation, this perspective article seeks to move beyond superficial comparisons of therapeutic methods by proposing a framework grounded in computational neuroscience and systems neurorehabilitation. We contend that Bobath-based handling modulates stretch reflex thresholds and reduces motor command noise, thereby refining internal state estimation. In parallel, task-oriented training updates motor policy through reward prediction errors linked to successful performance (19). Situating both within a dynamical systems perspective reveals that their potential synergy depends not on simple behavioral addition but on the induction of timing-dependent plasticity across cortico-reticulospinal-cerebellar circuits (20, 21) (Figure 1). This perspective article aims to establish a foundation for adaptive clinical trials stratified by neurophysiological biomarkers rather than behavioral labels alone. The ultimate goal is to enhance the precision and efficiency of upper limb recovery in the era of personalized neurorehabilitation.

## 2 Reconceptualization: from methodological dichotomy to a temporal coupling perspective

To enhance conceptual coherence, the following sections examine the proposed framework across complementary levels of analysis. We first consider the neurophysiological modulation of sensorimotor variability. Next, we examine reinforcement-based mechanisms underlying use-dependent plasticity. These components are then integrated into a unified temporal coupling framework. This integration provides a system-level account of their interaction.

### 2.1 Neurophysiological reappraisal of the contemporary Bobath concept

The contemporary Bobath concept extends well beyond the traditional goal of global tone inhibition (22). Current neurophysiological interpretations emphasize its role in recalibrating anticipatory postural adjustments and selectively reweighting somatosensory afferent input (23). The central mechanistic hypothesis proposes that skilled manual guidance functions primarily as a process of noise pruning within the sensorimotor loop. Through precisely modulated peripheral input—specifically targeting muscle spindle afferents and cutaneous mechanoreceptors—the intervention transiently regulates the excitability of spinal premotor interneuron pools (24). This extrinsic modulation reduces the variance of motoneuron recruitment patterns and diminishes the entropy inherent in pathological muscle co-activation, thereby contributing to a more stable preparatory motor state.

To clarify the hierarchy of mechanisms proposed in this framework, sensorimotor noise pruning is assigned the primary functional role among the effects of therapist-guided facilitation. The reduction of motor variability is hypothesized to establish a more stable neural operating state (25, 26). Secondary consequences—including modulation of corticospinal excitability, stabilization of sensorimotor predictions, and enhanced temporal coordination across distributed neural populations—are therefore interpreted as mechanistic extensions of this same foundational process rather than as independent explanatory models (26, 27).

Importantly, this interpretation of therapist-guided handling as a form of sensorimotor noise pruning should currently be viewed as a conceptual and operational framework rather than a directly validated neurophysiological mechanism. Although indirectly supported by studies on movement variability, proprioceptive modulation, and motor coordination stability, direct experimental evidence remains limited—particularly regarding whether Bobath-based facilitation reduces entropy within neural motor ensembles (28, 29). Consequently, the present framework is primarily intended as a hypothesis-generating model to guide future empirical investigation.

### 2.2 Synaptic encoding logic of task-oriented training

Task-oriented training operates through the established mechanisms of use-dependent plasticity and reinforcement learning (30, 31). Its therapeutic efficacy depends less on the sheer volume of repetitions than on the temporal coupling between successful task completion and phasic dopaminergic release (32). When a motor attempt yields a positive reward prediction error—meaning the sensory outcome exceeded internal expectations—the resulting neuromodulatory signal stabilizes and potentiates the active synaptic ensemble (32). A critical caveat, however, remains. If the motor commands executed during these trials are characterized by high-dimensional noise and aberrant temporal sequencing, synaptic consolidation will inadvertently reinforce a maladaptive motor engram (33, 34).

### 2.3 Core hypothesis: temporal coupling within neural ensembles

We define temporal coupling as the precise, spike-timing-dependent alignment of descending cortical commands with spinal motoneuron recruitment and subsequent muscle activation (35). To enhance conceptual clarity and empirical testability, temporal coupling in this framework can be further operationalized as a reduction in latency variability between central motor output and peripheral muscle activation (36, 37). In experimental settings, this construct may be approximated using quantitative neurophysiological metrics, including high-density surface electromyography-derived activation timing variability, inter-muscle coherence, and spike-triggered averaging techniques (37, 38).

To bridge the gap between therapist-guided physical interaction and the molecular realm of synaptic plasticity, we posit that manual facilitation first engages a set of mesoscopic neurophysiological processes (39). Specifically, it may modulate sensorimotor oscillatory activity, reorganize mu- and beta-band synchronization patterns, and recalibrate corticomuscular coherence (40, 41). These intermediate phenomena provide a mechanistic translation pathway: they convert macroscopic improvements in movement organization into neural states marked by heightened temporal consistency and an increased receptivity to activity-dependent plasticity (39, 42). Although direct empirical evidence for these links remains sparse, such biomarkers offer a physiologically coherent bridge from behavioral intervention to downstream synaptic adaptation (40, 43).

The logical derivation of this framework proceeds in two stages. First, if the intrinsic motor output contains temporally disorganized synergistic noise, then repetitive task practice will strengthen these erroneous timing codes through Hebbian mechanisms, thereby consolidating compensatory rather than restorative patterns. Second, the preparatory application of the Bobath concept is hypothesized to establish a low-entropy motor baseline (23). By reducing trial-to-trial variability in muscle activation latencies and constraining kinematic degrees of freedom, this phase enhances the signal-to-noise ratio of the ensuing voluntary command (44). We therefore propose that this sequential interaction—noise pruning followed by reward-based reinforcement—constitutes a necessary neurophysiological precondition for driving restorative neuroplasticity within the lesioned hemisphere (Figure 1).

## 3 Biological evidence and logical deduction of synergistic interaction

Building on the preceding sections, this part does not reiterate individual theoretical frameworks. Instead, it integrates them into a unified systems-level interpretation. The aim is to synthesize neurophysiological modulation, predictive coding, and reinforcement learning mechanisms into a coherent account. This account explains how temporally structured interventions may influence motor recovery.

### 3.1 Module one: postural control as the spatiotemporal substrate for task execution

Skilled upper limb movement depends fundamentally on a stable postural scaffold (45). The contemporary Bobath concept emphasizes proximal stabilization and core control, mediated in part by descending modulation of the reticulospinal tract (21). This bilateral pathway regulates axial and proximal musculature, dampening postural sway and reducing the interference that instability imposes on distal precision (21, 46). By stabilizing the trunk and shoulder girdle, the intervention frees attentional and cortical resources within a bandwidth-limited neural system. Proximal stability therefore serves as an essential spatiotemporal substrate for dexterous grasp (47).

### 3.2 Module two: somatosensory predictive coding and internal model refinement

Manual guidance in Bobath-based handling can be understood through the lens of predictive coding and active inference (48). The therapist’s key points of contact provide a constrained, error-minimizing prior for the intended movement trajectory (48). In this formulation, the therapist’s hands function as an external Bayesian prior, supplying the lesioned brain with a reliable somatosensory template (49, 50). This process reduces prediction error and stabilizes sensory expectations. By attenuating prediction error during the preparatory phase, the system enters a state of heightened perceptual certainty (49). Notably, this predictive coding mechanism does not constitute a substitute for reinforcement-based learning; rather, it serves a complementary role by structuring the internal neural state upon which subsequent reward-driven plasticity operates. This modulation may enhance the effectiveness of subsequent learning processes (48, 51).

Nevertheless, the notion that therapist-guided somatosensory input stabilizes internal predictive models and thereby promotes downstream plasticity remains inferential (23, 52). Current evidence more robustly substantiates the general principles of predictive coding and sensorimotor integration than it validates the specific mechanistic pathway proposed here (49, 53). Accordingly, these interpretations should be regarded as theoretically informed extrapolations rather than definitive causal explanations.

### 3.3 Module three: the gating hypothesis of spike-timing-dependent plasticity

The significance of a single, high-quality joint alignment achieved through Bobath guidance lies not in the movement itself but in its potential to promote temporally coordinated neuronal activity (54). When therapist-guided movement facilitates alignment between corticospinal output and task-relevant spinal interneuron activity, it may transiently increase the coherence of population-level neural firing patterns (54). Within this context, we propose that the preparatory phase may function as a permissive gating process for spike-timing-dependent plasticity (STDP), by biasing the system toward more temporally structured activation states.

Importantly, STDP should not be conceptualized as an isolated or purely timing-driven mechanism. Its induction is critically modulated by the underlying physiological state of the neural system, including inhibitory–excitatory balance, prior synaptic activity, and neuromodulatory influences such as dopaminergic and cholinergic signaling (55, 56). Consequently, even when temporal alignment is improved, plasticity may not be expressed unless the system resides within a permissive state (55, 56). This highlights the necessity of incorporating state-dependent and metaplastic mechanisms into the proposed framework, thereby constraining the conditions under which timing-related interventions can effectively induce synaptic change.

Recent advances in neurorehabilitation research further suggest that the effectiveness of motor learning depends not solely on stimulation timing itself, but also on whether the nervous system is operating within a transient plasticity-permissive state (57, 58). Emerging evidence from state-dependent neuromodulation studies indicates that fluctuations in cortical oscillatory dynamics, inhibitory-excitatory equilibrium, and large-scale network synchronization may substantially influence the responsiveness of the motor system to rehabilitative input (59). In parallel, studies of metaplasticity indicate that prior neural activity continuously reshapes the threshold for subsequent synaptic modification, thereby influencing whether repetitive practice promotes adaptive functional reorganization or reinforces maladaptive motor patterns (60, 61). Collectively, these findings provide broader neurobiological support for the central premise of the present framework: namely, that the efficacy of rehabilitation may depend not only on the behavioral content of training, but also on the evolving internal neurophysiological conditions under which that training is delivered.

By biasing the network toward a more temporally organized activation pattern, the intervention may increase the probability—rather than guarantee—that subsequent voluntary motor attempts satisfy the pre-before-post conditions associated with long-term potentiation (54, 62, 63). In this sense, the proposed gating mechanism should be understood as a probabilistic facilitator of Hebbian-like learning processes, rather than a deterministic trigger. This perspective provides a more conservative and biologically grounded interpretation of how preparatory modulation and task-oriented reinforcement may interact to bias plasticity toward restorative rather than maladaptive motor representations.

## 4 Future paradigm: toward closed-loop, computational, and personalized neurorehabilitation

### 4.1 Critique of the prevailing paradigm

Current clinical practice typically employs a serial intervention model, wherein distinct therapeutic modalities are delivered in fixed temporal succession-for instance, 20 min of Bobath handling followed by 30 min of occupational therapy. While logistically practical, this compartmentalized approach creates a fundamental neurodynamic disconnect (50). The preparatory modulation of spinal and cortical excitability achieved during the initial phase inevitably diminishes before task-specific reinforcement commences (64). Consequently, the two interventions operate in relative neurophysiological isolation, squandering the transient window of heightened synaptic receptivity that justifies their sequential use (64, 65).

### 4.2 Proposed framework: a temporal coupling-based closed-loop system

We propose a next-generation rehabilitation architecture governed by the principle of state-dependent precision neuromodulation. Importantly, the present framework should not be interpreted as an immediately deployable clinical protocol or a fully mature technological platform. Rather, it is intended as a conceptual and hypothesis-generating model illustrating how the temporal coupling hypothesis could eventually be operationalized within future neurorehabilitation paradigms (57, 58). Accordingly, the purpose of this section is not to predict technological evolution itself, but to emphasize the neurophysiological importance of aligning therapeutic timing with dynamically fluctuating brain states (66, 67).

This closed-loop system integrates two functional modules. First, an assessment module employs high-density surface electromyography or wearable inertial sensors to compute real-time indices of muscle synergy temporal variability (68). When the system detects elevated entropy in muscle activation sequencing-indicating disorganized synergistic noise-it initiates a targeted somatosensory modulation protocol (68). This may involve vibrotactile feedback applied to key proprioceptive landmarks, therapist-guided sensorimotor facilitation, or future robotic and haptic assistance systems (69). Importantly, therapist-delivered interventions should be interpreted as mechanisms for modulating broader plasticity-permissive states rather than directly implementing millisecond-scale STDP (39, 70). Achieving the precise temporal alignment required for STDP would more plausibly require automated interfaces, adaptive neurostimulation systems, or robotic platforms capable of real-time closed-loop control (71, 72).

Importantly, beyond quantifying temporal coherence in motor output, the system should also incorporate indicators of the underlying neural state to more accurately guide intervention timing. Potential markers may include cortical excitability, oscillatory phase dynamics, or other neurophysiological signatures reflecting the readiness of the system to undergo plastic change (73, 74). Integrating such state-sensitive measures would allow the framework to identify whether the sensorimotor network is operating within a plasticity-permissive window, rather than assuming that improved temporal organization alone is sufficient to induce plasticity (73, 74). This perspective is consistent with emerging evidence highlighting the regulation of motor learning windows and underscores the importance of aligning therapeutic interventions with the evolving internal state of the brain.

Second, once the temporal coherence of muscle recruitment enters an optimal low-variability range, the system immediately triggers high-intensity, task-specific challenges tailored to the patient’s current motor capacity (75, 76). This coordinated transition is intended to ensure that reinforcement learning processes are engaged under conditions that are both temporally structured and biologically permissive, thereby potentially enhancing the specificity and efficiency of adaptive neuroplasticity.

### 4.3 Technological outlook and validation pathways

Emerging neuroimaging and computational tools offer promising pathways for empirical validation of the temporal coupling hypothesis (77). Several candidate biomarkers appear particularly relevant for this purpose. At the cortical level, oscillatory power in the mu (8–13 Hz) and beta (13–30 Hz) frequency bands, the magnitude and time course of event-related desynchronization, and the strength of corticomuscular coherence may collectively reflect the temporal organization of sensorimotor processing (78–80). At the peripheral level, high-density electromyography provides a means to quantify latency variability in muscle activation, the degree of motor unit synchronization, and the stability of inter-muscle coordination (81). The present framework predicts that successful rehabilitation will be characterized by progressive reductions in activation timing variability together with enhanced corticomuscular coherence. Notably, these predictions diverge from conventional models that emphasize cumulative training dose or behavioral repetition as primary drivers of recovery; instead, the framework anticipates measurable improvements in temporal coordination metrics that precede overt functional gains.

Concurrent fNIRS-EEG monitoring can quantify functional connectivity between prefrontal executive regions and primary motor cortex, providing real-time guidance on the appropriate intensity and directional vector of manual facilitation (82, 83). Additionally, patient-specific digital twins (computational models that integrate individualized musculoskeletal geometry with neural control parameters) enable simulation and optimization of temporal pairings between manual guidance and task execution (84, 85). These *in silico* simulations generate testable hypotheses regarding intervention timing, informing adaptive clinical trial designs that refine therapeutic parameters with greater precision (77). At present, however, these approaches remain largely exploratory and should be viewed primarily as methodological thought experiments to support future validation of the temporal coupling framework (66, 67). Their inclusion is intended to illustrate possible translational directions, not to imply current clinical readiness or established efficacy.

From a translational perspective, the implementation of these measurement and modeling approaches requires careful consideration of the balance between technical precision and clinical feasibility (86, 87). Scalable deployment will depend on the use of lightweight wearable sensors, streamlined signal-processing pipelines, and seamless integration into existing rehabilitation workflows (86–88). Such design considerations are essential to ensure that these technologies can be adopted beyond specialized research environments and translated into routine clinical practice (86–88). Ultimately, this integrative platform aims to transform neurorehabilitation from a standardized, protocol-driven practice into an adaptive, computationally informed discipline tailored to each patient’s unique neurophysiological profile.

## 5 Discussion and challenges

### 5.1 Practical barriers to clinical implementation

Translating the proposed temporal coupling framework into routine practice faces a significant barrier: the heterogeneity of therapist manual expertise (89). Its effective delivery depends upon the practitioner’s haptic sensitivity, perceptual attunement to subtle changes in muscle tone, and capacity for real-time adaptive modulation of afferent input (22, 89). This variability introduces substantial uncontrolled variance, complicating both clinical replication and empirical validation (90).

A critical step toward addressing this variability and advancing clinical translation is the establishment of quantifiable metrics that capture the constructs of “noise reduction” and “timing precision” fundamental to the framework (91). Candidate indicators include the temporal consistency of muscle synergy activations extracted from high-density electromyography, the inter-joint coordination stability quantified through inertial measurement units, and the trial-to-trial latency variability in movement initiation (92, 93). These parameters operationalize the theoretical benefits of optimized afferent input into empirically testable variables (91). Encouragingly, the growing availability of wearable sensor arrays and portable electromyographic systems provides technically feasible tools for capturing such metrics in ecologically valid clinical environments, thereby bridging the gap between controlled laboratory paradigms and the complexities of routine therapeutic settings (91). Accordingly, a key priority is to develop standardized yet flexible metrics. These metrics should be capable of quantifying intervention fidelity while preserving individualized responsiveness.

### 5.2 Boundary conditions of the temporal coupling hypothesis

The proposed framework may not be universally applicable across all stroke populations. Its effectiveness likely depends on the preservation of a minimum level of residual neural connectivity and adaptive plastic potential (94). Patients with extensive corticospinal tract disruption, severe fixed spasticity, profound sensorimotor deafferentation, or advanced structural degeneration may be unable to reliably enter the plasticity-permissive states assumed by the model (94, 95). Under such circumstances, improvements in temporal organization may be insufficient to support meaningful restorative plasticity. Consequently, future studies should explicitly investigate neuroanatomical and neurophysiological boundary conditions—including corticospinal tract integrity, cortical excitability profiles, and large-scale network connectivity—to determine which patient subgroups are most likely to benefit from temporal coupling-based interventions.

### 5.3 Rethinking evidence-based methodology

The hypothesis advanced here carries important implications for clinical trial design in neurorehabilitation. Conventional randomized controlled trials, which compare aggregated group means across fixed intervention arms, are methodologically ill-suited to detect temporally contingent, individualized effects (96, 97). The synergistic benefit of sequential Bobath priming followed by task-oriented training may emerge only when the interval between interventions falls within a narrow, patient-specific neurophysiological window (98, 99). Such nuanced, state-dependent phenomena are inevitably obscured by standard between-group comparisons (96, 100). We therefore advocate a methodological shift toward N-of-1 trial designs or adaptive frameworks such as sequential multiple assignment randomized trials (96, 100). These approaches permit rigorous evaluation of time-varying treatment sequences and enable identification of optimal temporal coupling parameters at the individual level, thereby aligning research methodology with the personalized, dynamic nature of the proposed mechanism.

### 5.4 Implications for neurorehabilitation education

The conceptual shift from methodological dichotomy to temporal coupling requires a corresponding transformation in professional education. The pedagogical tradition of presenting Bobath and task-oriented training as competing therapeutic schools is both scientifically outdated and clinically counterproductive (101, 102). This framing perpetuates a false binary that obscures the fundamental neurophysiological complementarity of the two approaches (9, 101, 102) (Table 1). Future curricula should instead emphasize a unified framework centered on neurodynamically informed intervention timing. Trainees should learn to assess the fluctuating state of the patient’s sensorimotor system and to select the appropriate modality—whether afferent noise reduction or intensive task practice—based on real-time evaluation of movement quality and temporal coherence. Such an educational reorientation would cultivate clinicians capable of deploying therapeutic tools not as expressions of allegiance to particular brands of therapy, but as flexible, physiologically grounded responses to the evolving condition of the recovering nervous system.

## 6 Summary

This perspective article proposes temporal neural coupling as a unifying meta-theoretical framework for understanding advanced neurorehabilitation after stroke. We argue that the longstanding tension between the Bobath concept and task-oriented training does not stem from inherent incompatibility. Rather, it reflects suboptimal temporal sequencing and insufficient mechanistic stratification. Bobath-based handling functions as sensorimotor noise pruning, while task-oriented training provides reward-based synaptic reinforcement. Their synergistic potential depends on precise, millisecond-scale alignment between therapeutic input and the fluctuating internal state of the lesioned sensorimotor network. This framework suggests that future breakthroughs in neurorehabilitation will not arise from inventing new therapeutic techniques or branded approaches. Instead, progress will depend on deploying neuroengineering tools—such as high-density electromyography, multimodal neuroimaging, and closed-loop feedback systems—to reveal and modulate the dynamic interaction between external interventions and the recovering brain. Precision neurorehabilitation therefore requires a fundamental shift: from rigid adherence to standardized protocols toward real-time, state-dependent orchestration of plasticity tailored to each patient’s unique recovery trajectory.

## Data Availability

The original contributions presented in the study are included in the article/supplementary material, further inquiries can be directed to the corresponding author.
